# The role of the South Pacific in modulating Tropical Pacific variability

**DOI:** 10.1038/s41598-019-52805-2

**Published:** 2019-12-04

**Authors:** Christine T. Y. Chung, Scott B. Power, Arnold Sullivan, François Delage

**Affiliations:** 1 Bureau of Meteorology, Melbourne, Australia; 2 CSIRO Marine and Atmospheric Research, Aspendale, Australia

**Keywords:** Atmospheric dynamics, Physical oceanography

## Abstract

Tropical Pacific variability (TPV) heavily influences global climate, but much is still unknown about its drivers. We examine the impact of South Pacific variability on the modes of TPV: the El Niño-Southern Oscillation (ENSO) and the Interdecadal Pacific Oscillation (IPO). We conduct idealised coupled experiments in which we suppress temperature and salinity variability at all oceanic levels in the South Pacific. This reduces decadal variability in the equatorial Pacific by ~30% and distorts the spatial pattern of the IPO. There is little change to overall interannual variability, however there is a decrease in the magnitude of the largest 5% of both El Niño and La Niña sea-surface temperature (SST) anomalies. Possible reasons for this include: (i) reduced decadal variability means that interannual SST variability is superposed onto a ‘flatter’ background signal, (ii) suppressing South Pacific variability leads to the alteration of coupled processes linking the South and equatorial Pacific. A small but significant mean state change arising from the imposed suppression may also contribute to the weakened extreme ENSO SST anomalies. The magnitude of both extreme El Niño and La Niña SST anomalies are reduced, and the associated spatial patterns of change of upper ocean heat content and wind stress anomalies are markedly different for both types of events.

## Introduction

Tropical Pacific variability (TPV) is a major influence on global climate through processes like the El Niño Southern-Oscillation (ENSO^1,2^) and the Interdecadal Pacific Oscillation (IPO^3,4^). On interannual and multi-year time-scales, ENSO has a direct impact on both seasonal and extreme temperature, sea level, and precipitation levels across the world^1,5–8^. On decadal and multi-decadal time-scales, the IPO has been shown to impact global mean surface temperature trends^9–11^, with remote effects on surface temperature and sea ice trends evident as far as Antarctica and the Arctic^12–15^.

There has been a large amount of research focused on the complex origins of TPV. While ENSO likely originates locally, the decadal component of TPV is associated with multiple modes and is enhanced by stochastic ocean-atmosphere feedbacks^16,17^. There also exists an important coupled feedback loop between the tropics and extratropics which plays an important role in modulating Pacific variability at various timescales^18,19^. Sub-seasonal to seasonal changes in the tropical Pacific SSTs can drive changes in the Hadley and Ferrell cells, which in turn influence the zonal wind anomalies and subtropical jets^18^. On decadal timescales, tropical SSTs can also drive changes in the Hadley cells, Aleutian low, and Southern Hemisphere pressure systems^19^. These tropical SST-driven changes in atmospheric circulation, in turn, drive a coupled response in extratropical ocean temperatures, salinity, and currents, thus are termed the ‘atmospheric bridge’^20^. In the Pacific, changes in the Hadley circulation induce wind-stress curl anomalies, affecting the North and South Pacific subtropical gyres near the surface, and the subtropical vertical cells in the subsurface. For example, on decadal timescales, through this coupled feedback loop, a cooling in tropical Pacific SSTs can drive a weakening of the Hadley circulation, which in turn drives a slowdown of the subtropical gyres and cells. This reduction in the subsurface meridional heat transport (termed the ‘oceanic tunnel’) then generates positive SST anomalies in the tropical SSTs^19^.

More recently, with the availability of more sophisticated climate models, it has been found that Tropical Pacific sea surface temperatures are also likely influenced by variability in remote oceanic regions^21^. There is evidence that TPV is linked to stochastic atmospheric forcing from the South Pacific^22^, and that climate models which have less subsurface variability in the Southern Tasman Sea exhibit less decadal variability in the Niño3.4 region in the central equatorial Pacific^23^. The North and South Pacific Meridional Modes (NPMM, SPMM)^24,25^ have also been identified as important drivers of TPV on multi-year to decadal timescales^26^. The influence of the Atlantic Multidecadal Oscillation (AMO) on the Pacific has also been well documented^27–29^, with the AMO modulating the Walker circulation, leading to modified wind anomalies over the equatorial Pacific^27^. In the Western Tropical Pacific, multidecadal variability may even be more strongly influenced by the AMO than the IPO^28^.

In this study, we focus on the role of the South Pacific in modulating the naturally-occurring, internally-generated variability in the Tropical Pacific. To achieve this, we run a ‘modified pacemaker’ experiment using the Australian Community Climate and Earth System Simulator (ACCESS-CM2j) model (see Methods for model description and detailed experimental setup). We compare several hundred years of a control simulation (called the CTL run), run under pre-industrial conditions, to a simulation in which temperature and salinity in all ocean depths in the South Pacific (30°S–10°S, 165°W–85°W) are “clamped”, or nudged, back to the model’s climatological values (called the STHPAC run). In this way, any year-to-year variability in the region is suppressed. Similar experiments have been performed previously by authors who clamped the SST in slightly different regions in the South Pacific^16,26^. The first group investigated the effect of clamping tropical (20°S–20°N) and extratropical SSTs (20° to poles) in separate 150-year long runs^16^. The authors found that clamping tropical SSTs had a small impact on North Pacific variability, and that clamping extratropical SSTs reduced tropical Pacific decadal variability by approximately 25%. They also performed an experiment in which they clamped temperature and salinity at all levels in the South Pacific, which reduced decadal variability in the tropical Pacific by up to 30%. The second group performed experiments in which they clamped SSTs to observed 1920–2005 climatological values over the regions where the North and South Pacific Meridional Modes are most active (in the north-eastern and south-eastern Pacific respectively), located in the eastern part of the North and South Pacific respectively^26^. They found that clamping the North Pacific Meridional Mode region reduced the interannual TPV by ~35% but had little impact on decadal TPV, whereas clamping the South Pacific Meridional Mode region reduced only the decadal TPV by ~30% and had no impact on interannual TPV.

We extend this previous work in the following ways: (i) By performing an extended run (400 years) we are able to capture more of the model’s low-frequency variability. (ii) By clamping the temperature and salinity at all levels instead of just at the surface, and using a newer, higher-resolution model, we are able to better investigate the impact of disabling the ‘oceanic tunnel’, in addition to disabling the ocean-atmosphere coupling over the South Pacific. As both temperature and salinity determine the density gradient of the ocean^30^, clamping both temperature and salinity allows us to significantly dampen oceanic variability in the South Pacific. (iii) By running the experiments under pre-industrial control conditions and nudging the model to its own climatology, we can isolate the effect of internally-generated variability and avoid potential discrepancies in temperature patterns due to model bias.

## Results

### Modelled climatology, climate drift and associated mean state changes

**Figure 1:**
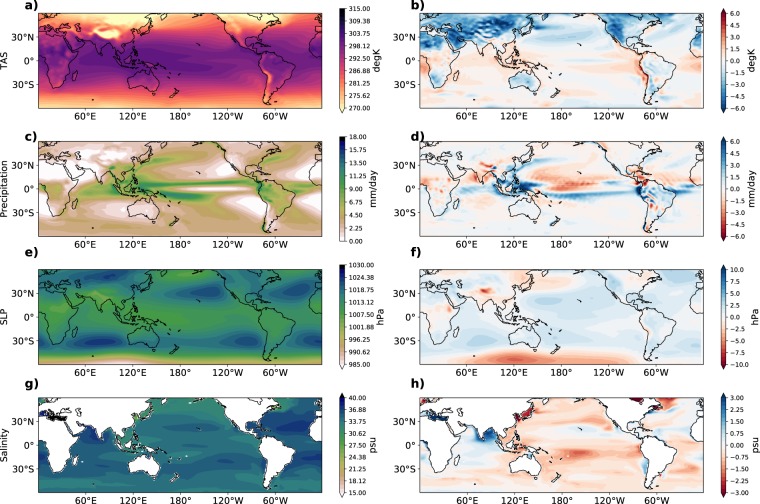
Comparison of modelled and reanalysis climatologies. Climatological values from the control run (left column) and corresponding biases with respect to reanalyses (right column) for (**a,b**) temperature at 1.5 m, (**c,d**) precipitation, (**e,f**) sea level pressure and (**g,h**) sea surface salinity. Details of reanalysis datasets are in the text.

Before presenting the results of the experiments, we first discuss some aspects of the ACCESS-CM2j model’s climatology, drift, and the mean state change from the experiment. We check the model’s surface air temperature, precipitation, and sea level pressure climatology (Fig. 1a,c,e) against years 1850–1900 of the National Oceanic and Atmospheric Administration-Cooperative Institute for Research in the Environmental Science (NOAA-CIRES) 20th Century Reanalysis V2c^31^ (Fig. 1b,d,f). The climatology of the ocean salinity at 5 m (Fig. 1g) is compared to years 1900–1950 of the CMCC Historical Ocean Reanalysis (CHOR)^32^ (Fig. 1g). These reanalysis time periods are chosen so that they are as close to preindustrial conditions as possible. We note that this is a somewhat qualitative comparison as the CTL run is fixed at preindustrial conditions and does not simulate time-varying aerosols or greenhouse gases. In all four variables, the main climatological features each variable are simulated well. The surface air temperature (TAS) bias is less than 2 degrees in most regions, though there is a larger cool bias in the Northern Hemisphere. For example, for rainfall (Fig. 1c,d), the Intertropical Convergence Zone (ITCZ) and South Pacific Convergence Zone are present, though the model suffers from the common ‘double ITCZ’ bias^33^ and is too dry over the central equatorial Pacific. In mean sea level pressure (SLP; Fig. 1e,d), the semi-permanent high pressure systems in the North and South Pacific, North and South Atlantic, and South Indian Oceans are present. The salinity biases in the central Pacific and Indian Oceans (Fig. 1h) may be associated with the rainfall biases in those regions.

One potential issue affecting multi-century simulations is climate drift. Most models exhibit a spurious, systematic trend, or drift, in subsurface and surface temperatures^34,35^. To correct for this, we use the “full linear drift” method^35^ and subtract a linear trend from the full time series of the CTL and STHPAC runs. We note that the trend in global mean SST for the CTL run is relatively large (0.1 K per 100 years) and that clamping the South Pacific reduces this drift considerably to 0.004 K per 100 years. For reference, the CMIP5 multi-model mean SST drift is 0.02 K per 100 years. All results presented henceforth in this study have been linearly detrended at each grid point, with the CTL and STHPAC runs detrended separately.

**Figure 2:**
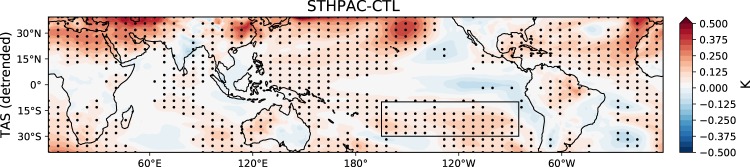
STHPAC-CTL mean state change in detrended surface temperature. The clamped region is outlined. Stippling indicates differences in the mean state significant at the 95% level.

Another issue we address is how much the mean states of the STHPAC and CTL simulations differ, and whether the mean state has changed significantly. In Fig. 2, we compare the average annual mean surface air temperature (TAS) from the detrended STHPAC and CTL runs. The map is stippled where the means of the two runs are significantly different at the 95% level. The STHPAC-CTL temperature difference exhibits an IPO-like structure, where the STHPAC run is warmer in the north, south, and western Pacific and slightly cooler in the eastern equatorial Pacific. Note that the lack of stippling in the central equatorial Pacific indicates there is no significant difference between the STHPAC and CTL climatologies in this region. In the rest of the following analysis, we use only the detrended data.

### Changes to tropical pacific SST variability

**Figure 3:**
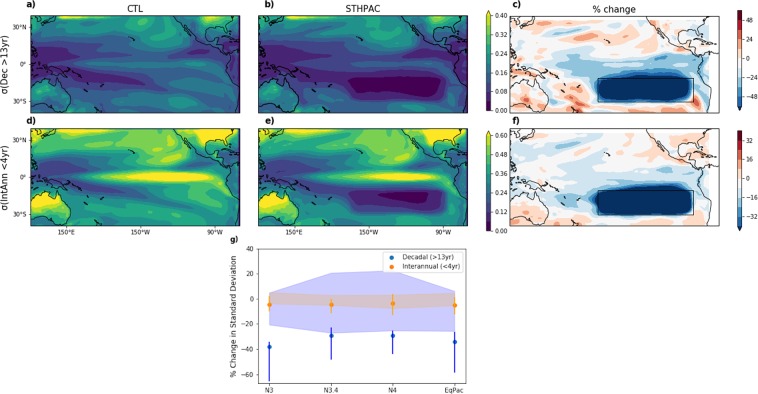
Effect of clamping South Pacific variability on Tropical Pacific variability. (**a,d**) Standard devation, σ, of surface temperature in the CTL run on (**a**) decadal, and (**d**) interannual timescales. (**b,e**) The same, but for the STHPAC run. (**c,f**) STHPAC – CTL percentage change in σ. (**g**) STHPAC – CTL percentage change in σ on interannual (orange) and decadal (blue) timescales, in the Niño 3, Niño 3.4, Niño 4, and equatorial Pacific regions. Shading indicates the spread of percentage change in σ from the four CTL ‘ensemble’ members, and vertical lines indicate the spread in the four STHPAC ‘ensemble’ members described in the text.

We now examine the effect of clamping the South Pacific on TPV on multiple timescales. Firstly, we compare the standard deviation of decadal (‘Dec’; low-frequency, >13 years) and interannual (‘IntAnn’; high-frequency, <4 years) time scales for the two runs. Figure 3(a–c) show the standard deviation in the 13-year lowpass filtered TAS for the CTL and STHPAC runs, σ(Dec), and the percentage change in σ relative to its CTL value. Figure 3(d–f) shows the same, but for 4-year highpass-filtered TAS, σ(IntAnn). As expected, in the clamping region the variability on both timescales decreases to almost zero. The decrease in variability also extends northwards, spanning the equatorial Pacific. On decadal timescales, there is an approximately 20% decrease in σ(Dec) in the central equatorial Pacific, flanked by a ~30% decrease in the eastern and western equatorial Pacific. On interannual timescales, this decrease is ~10% across the equatorial Pacific. To quantify this further, and to assess the significance of the change in several key regions, Fig. 3g shows the percentage change in standard deviation, σ, for the three Niño regions: Niño 3 (5°N–5°S, 150°W–90°W), Niño 3.4 (5°N–5°S, 170°W–120°W), and Niño 4 (5°N–5°S, 160°E–150°W), and for the equatorial Pacific (5°S–5°N, 120°E–300°E). On interannual timescales (orange markers), clamping the South Pacific causes an approximately 6% decrease in σ in all the regions. On decadal timescales (blue markers), σ decreases most in the Niño 3 region (38%), with a 29–30% decrease in both Niño 3.4 and Niño 4. In order to gauge the significance of the difference in these changes, we create a 4-member ‘ensemble’ by splitting up the 380 years of both CTL and STHPAC runs into four 95-year chunks. The spread of the percentage change in the CTL ensemble is indicated by the blue (decadal) and orange (interannual) shading, while the spread of the STHPAC ensemble is indicated by vertical lines. We find that the changes on interannual timescales are within, or close to, the orange shaded area, but the changes on decadal time scales fall well outside the blue shaded area. We now examine in more detail how clamping impacts the main modes of variability, the ENSO and the IPO.

### Changes to ENSO

**Figure 4:**
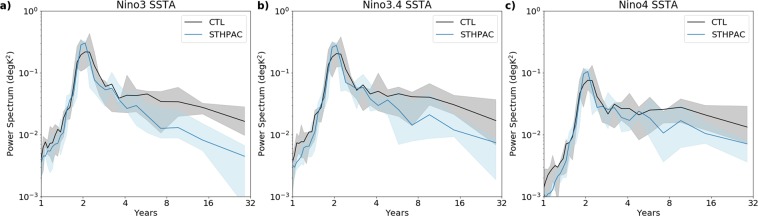
Power spectra of (**a**) Niño 3, (**b**) Niño 3.4, and (**c**) Niño 4 SST anomalies. Black line indicates the CTL run, and the blue line indicates the STHPAC run. The grey shading indicates the spread of the 4-member CTL ensemble, and the light blue shading indicates the spread of the 4-member STHPAC ensemble described in the text.

In the CMIP5 multi-model mean, ENSO SST anomalies extend too far west, and are too meridionally narrow^36^. The ACCESS-CM2j model simulates an ENSO SST pattern that does not extend as far west as the CMIP5 multi-model mean, representing an improvement in this regard. We note however, that the periodicity of ENSO is more biennial (i.e. has a 2-year periodicity) compared to the observed 3–4 year periodicity of ENSO. This is a common issue with CGCMs^37^ and the lack of persistence in ENSO SST anomalies can reduce the amount of decadal variability in such models. The power spectrum of Niño3, Niño3.4, and Niño4 indices (Fig. 4) shows that the CTL run (black line) peaks at 2 years, and that in the STHPAC run (blue line), the ENSO variability becomes even more biennial, with a narrower, higher peak at 2 years. There is also less power on the decadal time scales, consistent with the reduction of decadal variability along the equatorial Pacific seen in Fig. 3. The grey and light blue shading shows the range of power spectra produced by all four control ensemble members of the CTL and STHPAC runs respectively. In the Niño 3 and Niño 3.4 regions, the multi-year to decadal-scale (>8 years) STHPAC spectra lies outside the CTL ensemble shading, whereas in the Niño 4 region, the STHPAC spectrum overlaps with the shading at the 10–12 year timescale, but lies outside the CTL ensemble shading for >16 years.

**Figure 5:**
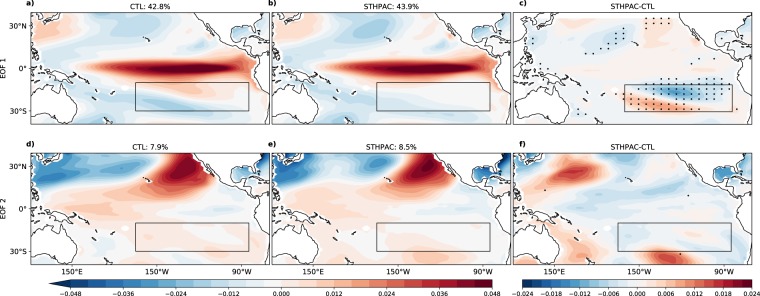
Changes to interannual-scale modes of variability such as ENSO. EOFs 1 (top) and 2 (bottom) of NDJF SST anomalies for the CTL (**a,d**) and STHPAC (**b,e**) runs, and the difference STHPAC – CTL (**c,f**). Stippling indicates where the magnitude of variability lies outside the range of the EOFs of the four CTL ensemble members.

As ENSO activity peaks in November-February (NDJF), we use only this season for the analysis for the rest of this subsection. The first two empirical orthogonal functions (EOFs) in tropical Pacific SSTs (Fig. 5), are associated with ENSO. Two types of El Niño events are commonly referred to, with the spatial pattern of EOF 1 resembling the Eastern Pacific (EP), or Cold Tongue (CT) El Niño^38–41^, and EOF 2 resembling the Central Pacific (CP), or Modoki, El Niño^39,41^. However, recent work has shown that both modes are important in distinguishing between the two types of El Niño events^42,43^. In the model, the first, dominant mode explains most of the variance from the central to eastern equatorial Pacific (Fig. 5a). We note that there is a bias in EOF 2 of the model, in which the tripolar pattern associated with the CP or Modoki El Niño^39,41^ is not well simulated. In EOF 2, the anomalous warming which occurs in the central equatorial Pacific in observations is located too far west in the model.

Comparing the STHPAC and CTL runs, EOF 1 remains mostly unchanged (Fig. 5a–c). Stippling in Fig. 5c,f indicate where the magnitude of the variability lies outside the range of the EOFs of the four ensemble members. Some differences are seen in the box where clamping occurs and the north Pacific, but the clamping in the South Pacific does not largely impact the rest of the tropical Pacific. This supports the theory that most variability in the equatorial Pacific is locally generated, and that it is not a response to variability from the sub-tropics. In the CTL run, EOF 1 accounts for 42.8% of the total variability, whereas in the STHPAC run, it is 43.9%.

Meanwhile, EOF 2 has a meridionally wider ‘boomerang’ pattern, with negative anomalies in the eastern equatorial Pacific flanked by positive anomalies to the north, west, and south extending into the clamping region. In the STHPAC run, the variability associated with EOF 2 in the clamped region and eastern equatorial Pacific is largely suppressed, resulting in the southern ‘arm’ of the boomerang being shifted to the south-west (Fig. 5d,e), although the lack of stippling in this region shows that the magnitude of the variability change lies within the ensemble spread (Fig. 5f). The stippling in the western Pacific indicates increased variability north of ~15°N, and decreased variability from 0–15°N. EOF 2 accounts for 7.9% and 8.9% of total variability in the CTL and STHPAC run respectively.

### Disruption of the IPO

**Figure 6:**
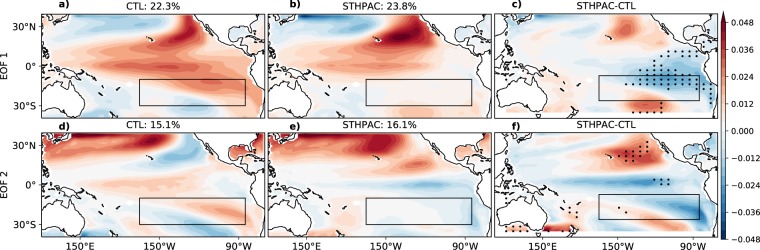
Changes to decadal-scale modes of variability such as the IPO. EOFs 1 (top) and 2 (bottom) of 13-year lowpass filtered SST anomalies for the CTL (**a,d**) and STHPAC (**b,e**) runs, and the difference STHPAC – CTL (**c,f**). Stippling indicates where the magnitude of variability lies outside the range of the EOFs of the four CTL ensemble members.

In observations, the IPO is defined to be the second EOF mode of lowpass-filtered Tropical Pacific SSTs, with the first mode corresponding to the global warming trend^3,44,45^. As our experiments are run under pre-industrial conditions, the IPO is defined to be the first EOF in the CTL and STHPAC runs. Figure 6 shows the first two EOFs of the 13-year lowpass filtered Tropical Pacific SST anomalies and the difference between them. The first EOF in the CTL run (Fig. 6a) represents the spatial structure of the IPO reasonably well and accounts for 22.3% of the variance. In the STHPAC run, EOF 1 constitutes 23.8% of the variance. However, as the spatial pattern of variability linked to the IPO pattern extends into the clamped region, the IPO pattern is altered (Fig. 6b). The difference between the STHPAC and CTL runs is shown in Fig. 6c and is stippled where the magnitude of the change is larger than the spread of the EOFs of the four ensemble members. The variability in the north-east Pacific is enhanced, while in the south-eastern Pacific, it is reduced.

The second EOF (constituting 15.1% in the CTL and 16.1% in the STHPAC runs) also exhibits notable spatial differences along the central and eastern Pacific. In the CTL run, the spatial pattern of EOF 2 (Fig. 6d) resembles the North Pacific mode, which is a multi-decadal mode of variability thought to be generated by stochastic atmospheric processes and ocean-atmosphere interactions in the North Pacific^46–48^. Although modes of decadal variability in the North Pacific are thought to be mostly locally generated, it has been shown that teleconnections from the tropical Pacific can impact variability in the north-eastern Pacific^49^. The STHPAC run exhibits a very different pattern in the central and eastern Pacific, which is another indication that South Pacific variability may play an important role in decadal-scale variability across the tropical Pacific.

### Distribution of ENSO events and impact on extremes

**Figure 7:**
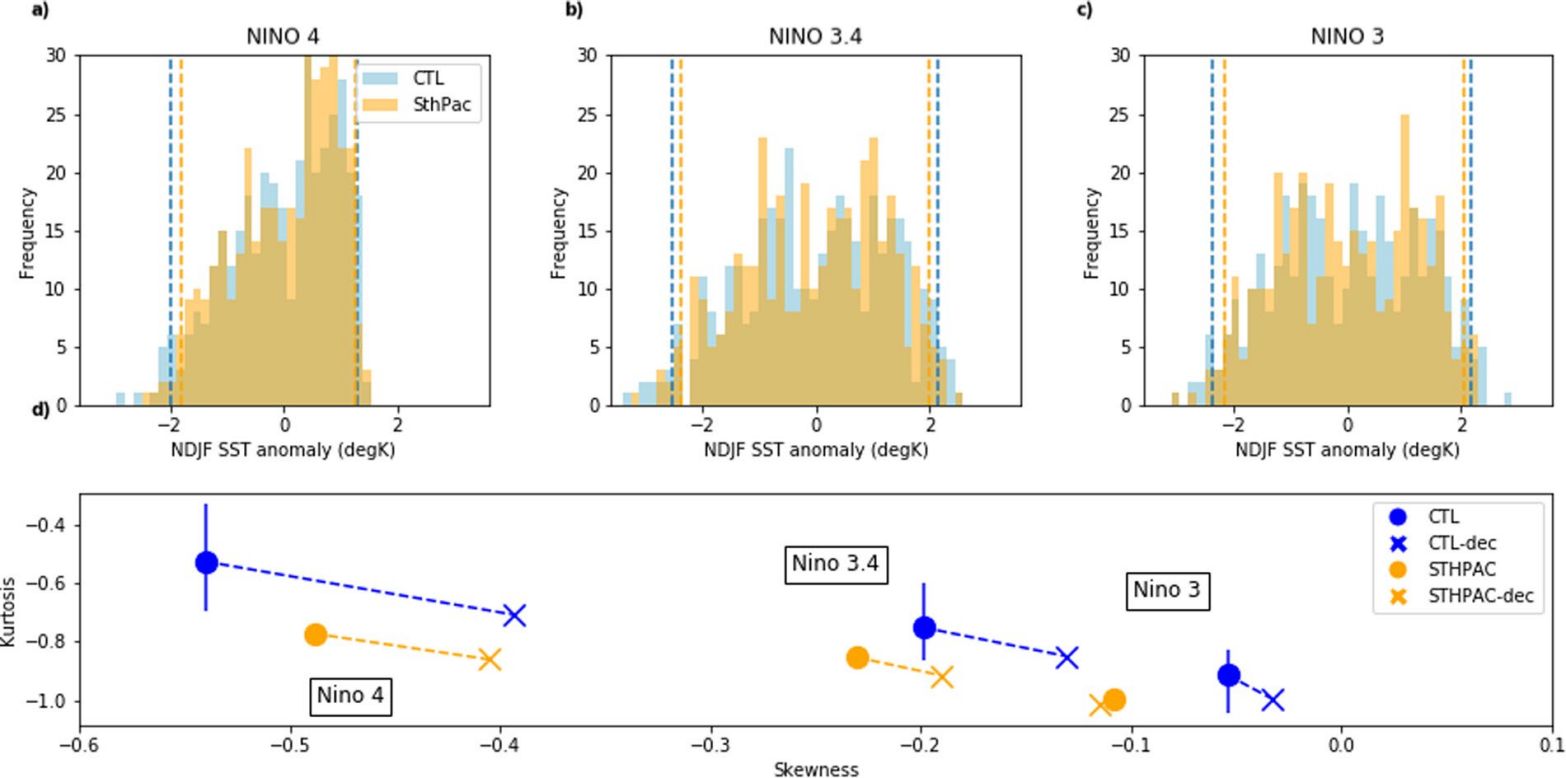
Changes to the distribution of SST anomalies in the Niño regions. Histograms of NDJF SST anomalies in the (**a**) Niño 4, (**b**) Niño 3.4, and (**c**) Niño 3 regions. CTL SST anomalies are shown in blue, while STHPAC SST anomalies are shown in orange. Dashed vertical lines indicate the largest 5% SST anomalies for each distribution. (**d**) Skewness-kurtosis plot for the SST distributions in (**a–c**). Blue circles correspond to the CTL run and orange circles correspond to the STHPAC run. The vertical blue lines indicate the spread of kurtosis values for the four CTL ensemble members. Blue and orange crosses correspond to the CTL and STHPAC runs respectively, *with the decadal signal removed* (CTL-dec and STHPAC-dec).

We have shown that suppressing variability in the South Pacific results in large changes in the amplitude and spatial patterns of decadal TPV. Although there is no significant change in the overall standard deviation of interannual TPV, we now examine the impact of clamping the South Pacific on the distribution of individual ENSO events. Figure 7(a–c) show the distribution of NDJF SST anomalies in the Niño 4, Niño 3.4, and Niño 3 regions for the CTL and STHPAC runs. In all the Niño regions, negative SST anomalies correspond to La Niña events, and positive SST anomalies correspond to El Niño events. The dashed lines indicate the largest 5% of positive and negative SST anomalies for each run, which we use as a threshold to distinguish ‘extreme’ ENSO events. Figure 7d shows a skewness-kurtosis plot for the distributions in Fig. 7(a–c) (see Methods section for definitions of skewness and kurtosis). The CTL and STHPAC runs are denoted by dark blue and orange circles respectively. Note the blue and orange crosses correspond to CTL and STHPAC runs *with decadal signal removed*, which we will discuss further below.

Looking first at the skewness, *s*, which measures the asymmetry of the distributions of SST anomalies (SSTAs), we note that the model is generally biased towards stronger La Niña events (negative skewness) compared to observations. For example, in the Niño 3 region, observed SST anomalies are positively skewed towards EN events (*s*_N3_OBS_ ~ 0.9^1^), however the modelled SST anomalies have weak negative skewness in both runs (Fig. 7c,d). Further west, as *s* becomes increasingly negative, the bias is still apparent, with *s*_N4_OBS_ ~ −0.5^1^ and *s*_N4_CTL_ ~ −0.54 in the Niño 4 region. However, in the STHPAC run, the westward shift towards negative skewness is reduced (*s*_N4_STHPAC_ ~ −0.49). It has been shown, in observed SSTs, the westward shift towards negative skewness is largely associated with extreme ENSO events, and without extreme events, the shift is reduced^1^. We now show that the change in *s*_N4_CTL_ and *s*_N4_STHPAC_ is also associated with a reduction in the SSTAs of extreme ENSO events.

Next, we examine the tails of the distributions, corresponding to extreme SSTAs (defined to be the largest 5% EN/LN events for each run). We note that the SSTA distributions in Fig. 7a–c show, on average, larger extreme SSTAs in the CTL run. Figure 7d shows that for all Niño regions, the STHPAC SST anomalies (orange circles) have a larger negative kurtosis, *k*, than the CTL (blue circles), indicating that the distribution of the STHPAC anomalies is more ‘light-tailed’, or has fewer outliers (see Methods section for a mathematical definition of kurtosis). The range of *k* values obtained from the four control ensemble members is indicated by vertical blue lines in Fig. 7d. The difference between the *k*__CTL_ and *k_STHPAC* is largest in the westernmost Niño 4 region, where *k*__STHPAC_ lies well outside the ensemble spread. In the Niño3 and Niño 3.4 regions, the *k*__STHPAC_ SSTAs lies within the ensemble spread. Note also that the STHPAC run, with weaker extreme events, exhibits an overall smaller westwards negative shift in skewness, consistent with previous research^1^.

**Figure 8:**
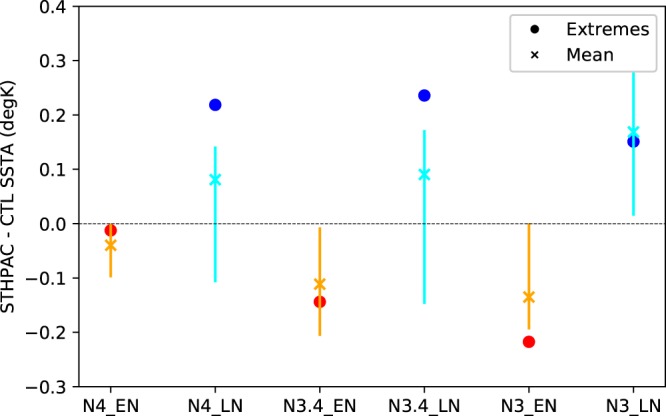
Mean STHPAC-CTL SST anomalies for extreme El Niño (red circles) and extreme La Niña (blue circles) events, and for all El Niño (orange crosses) and all La Niña events (cyan crosses). Positive blue and cyan values indicate CTL La Niña SST anomalies are stronger, and negative orange and red values indicate CTL El Niño SST anomalies are stronger.

To illustrate this further, in Fig. 8 we plot STHPAC-CTL SSTAs (δSSTAs) for all the Niño regions, for mean EN and LN events (orange and cyan crosses respectively), and for extreme EN and LN events (red and blue circles). Normal EN and LN events are defined to be those in which the SSTA exceeds one standard deviation of all SSTAs, and extreme EN and LN events are defined to be the largest 5% of EN/LN events for each run. The error bars show the spread of δSSTA for mean events from the four ensemble members. Figure 8 shows that in all the Niño regions, SSTAs from the CTL run are larger than those from the STHPAC run (i.e. δSSTA = positive for LN and negative for EN events). Additionally, in the Niño 3.4 and Niño 4 regions, the δSSTAs for extreme La Niña events (blue circles) lie well outside the ensemble spread for mean La Niña events (cyan crosses), indicating that in this model, clamping South Pacific variability impacts extreme La Niña events more than El Niño events in these regions. In the Niño 3 region, the δSSTAs for extreme El Niño events (red circles) lies outside the ensemble spread for mean El Niño events.

Figures 7 and 8 show that clamping the South Pacific reduces the strength of extreme ENSO events to some extent. This change in the properties of extreme ENSO events could be due to three possible reasons. The first arises from the fact that the decadal variability in STHPAC is weaker. If we suppose that the variability exhibited in the Niño indices arises from the linear superposition of a mid-to-high frequency (<13 years) and a lower frequency signal (>13 years), then the SST excursions in the Niño regions will tend to be reduced if the low-frequency signal is reduced. To test this hypothesis, we subtract the decadal signal, defined to be the 13-year lowpass filtered values, from the Niño indices. In doing this, the extreme SSTAs in both the CTL and STHPAC run are reduced (not shown). This is reflected in the reduced kurtosis values shown in Fig. 7d (blue and orange crosses, labelled CTL-dec and STHPAC-dec respectively). However, while subtracting the decadal signal does have an impact on extreme events, it does not explain entirely the difference between the CTL and STHPAC distributions in the Niño 3.4 and Niño 4 regions. The CTL-dec run, with decadal signal subtracted, still has stronger extreme events than the STHPAC run. This is evident in Fig. 7d, as the CTL-dec (blue crosses) kurtosis values are still larger than the STHPAC (orange circles) kurtosis values. Note also that subtracting the decadal signal reduces the negative skewness of the distributions in the Niño 3.4 and Niño 4 regions.

The second possibility arises as the variability in the South Pacific affects feedback processes connected with ENSO, for instance through the ‘atmospheric bridge/oceanic tunnel’ mechanisms^19,20^, or the SPMM^25,50^. The SPMM is linked to a coupled process in which equatorial Pacific SST anomalies are enhanced by the weakening of the trade winds inducing a deepening of the thermocline. In the months preceding an ENSO event, a sea level pressure anomaly is formed in the South Pacific^25^ in a region that overlaps with the clamped region in the STHPAC run. By decoupling the South Pacific, and weakening or disrupting these processes and teleconnections in the STHPAC run, we may disrupt some mechanism that contributes to the generation of extreme SSTAs. For example, in Supp. Fig. 1 we show the STHPAC-CTL SLP anomalies averaged over all LN and EN events, and for the largest 5% events. In many parts of the tropical Pacific, the change in SLP anomalies is approximately two times larger during extreme events. Thirdly, the mean-state change induced by clamping the South Pacific, discussed in Fig. 2, could also be a factor in the reduction of extreme SSTAs.

**Figure 9:**
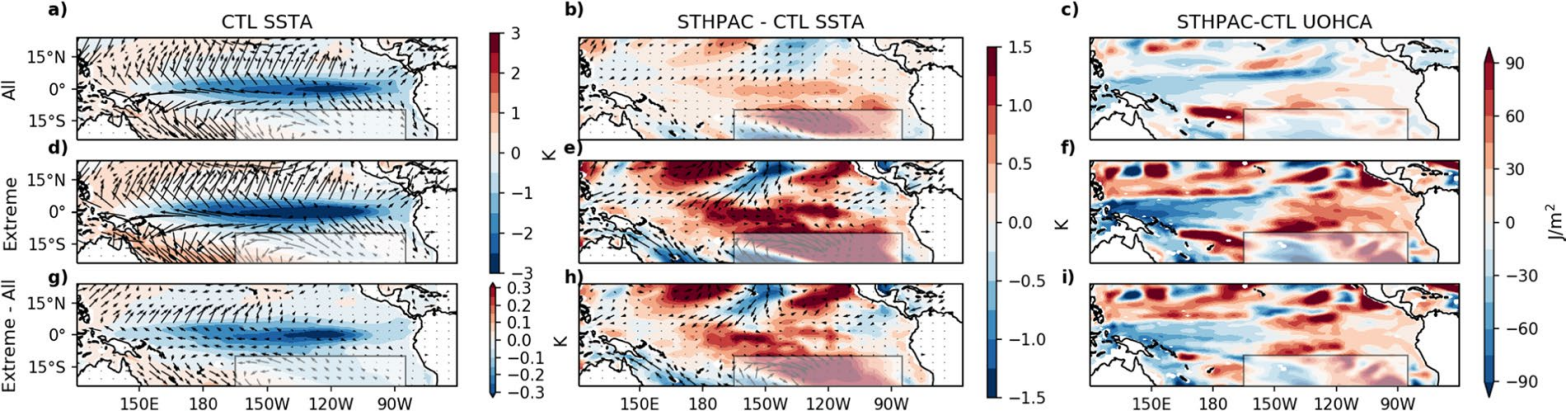
Changes to SST and upper ocean heat content anomalies under extreme and average La Niña conditions. (**a**) Mean CTL SST and wind stress anomalies for all (‘mean’) La Niña events, (**b**) STHPAC-CTL SST and wind stress anomalies for all La Niña events, (**c**) upper ocean heat content anomalies for all La Niña events. (**d–f**) The same as (**a–c**), but for the largest 5% (‘extreme’) of La Niña events (defined using Niño 3.4 SST anomalies). (**g–i**) The same as (**a–c**), but for extreme – mean La Niña events.

**Figure 10:**
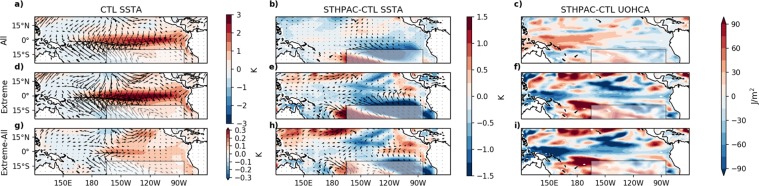
Changes to SST and upper ocean heat content anomalies under extreme and average El Niño conditions. The same as Fig. 9, but for El Niño events.

To investigate further the differences between the LN and EN events, in Figs 9 and 10 we compare various physical properties averaged over all LN and EN events, as well as averaged over the largest 5% of LN and EN events in the STHPAC and CTL runs (defined using Niño3.4 SSTAs defined with respect to each run’s own climatology). Looking first at LN events, the leftmost maps in Fig. 9 correspond to CTL LN SSTAs and wind stress anomalies all LN events, showing average LN conditions (top; Fig. 9a), the largest 5% LN (extreme) events (middle; Fig. 9d), and the difference between extreme and average conditions (bottom; Fig. 9g). The middle columns (Fig. 9b,e,h) show the same, but for the difference between STHPAC-CTL SSTAs (δSSTA). The rightmost columns (FCig. 9c,f,i) show the difference between the STHPAC and CTL upper ocean heat content anomaly (δUOHCA; temperature vertically integrated over the top 300 m). Under average LN conditions, along the equatorial Pacific, positive values of δSSTA in the central equatorial Pacific (Fig. 9b) indicate that the STHPAC run does not cool as much in this region during LN years, although the difference is relatively small (~0.5 K). In the STHPAC run, the δUOHCA (Fig. 9c) is negative in the western equatorial Pacific and positive in the east, indicating that the upper ocean does not warm as much in the west and does not cool as much in the east during LN years. Again, δUOHCA is small (~30 J/m^2^), however these small differences in δSSTA and δUOHCA are consistent with the ~6% reduction in interannual variability shown in Fig. 3d.

During the 10 largest LN events, when the SST anomalies are approximately 2 times larger than average, the SSTAs in the CTL run exhibit the largest decrease in the central-eastern Pacific between 120–150 W (Fig. 9g). However, the largest change in both δSSTA and δUOHCA occurs in the central Pacific between 150–180 W (Fig. 9h,i), with positive δSSTA and negative δUOHCA indicating that in the STHPAC run, the central Pacific does not cool as much as the CTL run during extreme LN events, resulting in a weaker zonal SST gradient, and weaker SSTAs, particularly in the Niño 3.4 and Niño 4 regions. This is consistent with weakened meridional wind stress anomalies originating from the Niño3.4 and Niño 4 regions in the STHPAC run, making the transport of heat away from the equator less efficient (Fig. 9h).

Figure 10 shows the same as Fig. 9, but for EN events. For average EN conditions (Fig. 10a–c), δSSTA and δUOHCA are approximately the inverse of average LN conditions (Fig. 9a–c), with negative δSSTA and positive δUOHCA in the central-eastern equatorial Pacific indicating that the STHPAC run warms marginally less in this region. However for the 10 largest EN events, we find differences in the spatial patterns of change compared to the extreme LN events. While the largest changes in the extreme LN events were centred on the Niño 3.4 and Niño 4 regions, for extreme EN events, we find the largest changes in δSSTA to be in the eastern equatorial Pacific between 90–120 W, corresponding to the Niño 3 region (Fig. 10e,h). Across most of the equatorial Pacific, δSSTA is also smaller for extreme EN events than for extreme LN events, although the overall effect is still a reduction of extreme SSTAs. This is also seen in Fig. 8, which shows that in the Niño 3.4 and Niño 4 regions, extreme EN δSSTAs are approximately 2 and 18 times weaker than the extreme LN δSSTAs respectively.

In the upper ocean, the spatial pattern of change in δUOHCA between extreme and average conditions is also markedly different for EN events. During extreme EN events, in the western equatorial Pacific, δUOHCA switches from being positive during mean EN events (Fig 10c) to being negative during extreme EN events (Fig 10f). This is accompanied by positive δUOHCA to the south-east around 150°W-180°W and 10°S, and negative δUOHCA to the north-east around 120°W-150°W and 10°N. The negative δUOHCA in the north is associated with weaker north-westerly surface wind stresses in the STHPAC run, whereas the positive δUOHCA in the south is associated with weaker south-westerly surface wind stresses.

As ENSO is such a highly coupled process, it is not straightforward to disentangle the changes in circulation and changes in oceanic processes induced by clamping the South Pacific. However these changes, combined with the reduction in decadal variability, act to reduce the amplitude of SST anomalies during extreme EN and LN events.

## Summary and Discussion

In summary, and taking the results at face value we conclude that clamping oceanic variability in the South Pacific causes:

i) a reduction in decadal-scale SST variability in the equatorial Pacific by ~30% which lies outside the ensemble spread,
ii) no significant change (i.e. the change lies within the ensemble spread) in high frequency (<4 years) SST variability in the equatorial Pacific,
iii) a decrease in the magnitude of extreme ENSO events, linked to a decrease in decadal variability, and the disruption of processes and teleconnections linking South Pacific variability to ENSO, and
iv) although the strength of both extreme EN and LN SST anomalies are reduced, the impact on LN events is larger, and the spatial patterns of change for the UOHC and wind stress anomalies are markedly different for both types of events.

Previous experiments which involved clamping South Pacific variability^16,26^ have also noted the ~30% decrease in decadal time-scale variability in the equatorial Pacific SST. However, this work provides new insight into the changes in interannual variability. While our finding that the ENSO-related modes of variability are not significantly changed by clamping the South Pacific is consistent with previous work, we have shown that the nature of *extreme* ENSO events may in fact change. The change in the strength of extreme ENSO-related SST anomalies shows that (i) the South Pacific provides a source of decadal variability that is necessary to facilitate large SST anomalies in the equatorial Pacific, and (ii) processes originating in the South Pacific may play a part in amplifying the SST anomalies in the equatorial Pacific.

Identifying the specific mechanisms which are responsible for the ~30% decrease in decadal variability remains an open question. However, the remarkable agreement between this study, in which we clamped South Pacific temperature and salinity variability at all ocean levels, and a previous study which clamped only South Pacific SST variability^26^, implies that it is coupled ocean-atmosphere processes (including the SPMM) that are primarily responsible for the reduction in decadal variability. There is also an interesting possibility that the reduction in the strength of extreme ENSO-related SST anomalies itself contributes to the reduction of decadal variability in the tropical Pacific. Another possibility is that the mean state change induced by clamping the South Pacific plays a role in the observed changes in both decadal variability and ENSO extremes. These will be explored in future experiments and analysis.

We note again that these results are subject to three model biases.. Firstly, as noted previously, the differences between the drift in the CTL and STHPAC runs may contribute to the differences in variability evident. Secondly, the degree of decadal variability in the model appears low, as the model is very biennial. Thirdly, the distribution of SST anomalies during ENSO events is skewed towards stronger La Niñas, contrary to observations. However, we also note that the results are consistent with experiments run using other models^16,26^. It is important to note also that we have only used a single model with a 380-year control simulation and a 380-year long perturbed integration. It would therefore be prudent to regard our findings as hypotheses for further research using more ensemble members and more models.

As we have shown, although much of the interannual variability in the Tropical Pacific is locally generated, the South Pacific may play an important role in modulating this variability. Recent research has also shown that teleconnections from the Atlantic and Indian Oceans also impact TPV on multiple timescales^21^. Experiments in which variability in the Tropical Atlantic and Indian Oceans are clamped are currently underway.

## Methods

### Model description

In this study we use the ACCESS-CM2j model^51^ which comprises the following. The atmospheric component (Unified Model GA7.1) atmospheric model component has 85 levels extending to 85 km, with a horizonal resolution of 1.25 × 1.875 in latitude and longitude, respectively^52^. The atmospheric CM2j refers to the second version of coupled model with the JULES land surface scheme. The oceanic component, MOM5^53,54^, has 50 unevenly spaced vertical levels and a latitude-longitude grid with a 1 degree meridional-direction grid spacing, with equatorial refinement to 1/3 degrees between 10°S–10°N to better resolve predominantly zonal equatorial ocean currents. The LANL CICE5.1.2 sea ice model version^55^ is used as the sea ice component of ACCESS-CM2j and is coupled to the ocean and atmosphere via the OASIS3-MCT coupler^56,57^.

A 400-year control run (called CTL) using pre-industrial conditions is used. Under pre-industrial conditions^58^, CO_2_, other greenhouse gases, and aerosols are set to constant pre-1850 values, and there is no external forcing applied. This allows the model to simulate a climate in which only internally-generated variability occurs.

To investigate the impact of South Pacific variability on Tropical Pacific variability, we run an additional 400-year simulation (called STHPAC) in which temperature and salinity at all levels in the South Pacific (30°S–10°S, 195°E–275°E) are nudged to the mode’s climatological values. The nudging occurs on a time scale of 5 days, and the climatology used is the mean of years 50–100 of the CTL run. The first 20 years of each run is discarded, leaving us with 380 years. To minimise discontinuities at the boundaries of the South Pacific box, we linearly decrease the damping coefficient to zero from the outer 5 degrees of each boundary (e.g. from 15°S–10°S in the north, 30°S–25°S in the south, 190°E–195°E in the west, and 270°E–275°E in the east).

### Skewness and Kurtosis

Here we briefly define skewness and kurtosis, used to describe the SSTA distributions in Fig. 7. The skewness of a distribution is its third standardised moment, and is a measure of its asymmetry. It is defined to be [Σ(X − X_m_)^3^/N]/σ^3^, where X_m_ is the mean, N is the size, and σ is the standard deviation of the distribution.

The kurtosis of a distribution is its fourth standardised moment, and is a measure of its “tailedness”, or the frequency of and amplitude of its outliers, relative to a normal distribution. It is defined to be [Σ(X − X_m_)^4^/N]/σ^4^.

## Supplementary information

Supplementary Figure

## Data Availability

All model output is stored on the National Computational Infrastructure and can be obtained by contacting Christine Chung (christine.chung@bom.gov.au).
