# Supplementary material for: The role of the South Pacific in modulating Tropical Pacific variability
Source: Sci Rep. 2019 Dec 4;9:18311. doi: 10.1038/s41598-019-52805-2 (PMC6892857; doi:10.1038/s41598-019-52805-2)
Supplement: Supplementary file 1 — Supplementary Figure [file 41598_2019_52805_MOESM1_ESM.docx]

The role of the South Pacific in modulating Tropical Pacific variability

Supplementary Information

Christine T. Y. Chung^1^, Scott B. Power^1^, Arnold Sullivan^2^, and François Delage^1^

*^1^Bureau of Meteorology, Melbourne, Australia*

*^2^CSIRO Marine and Atmospheric Research, Aspendale, Australia*


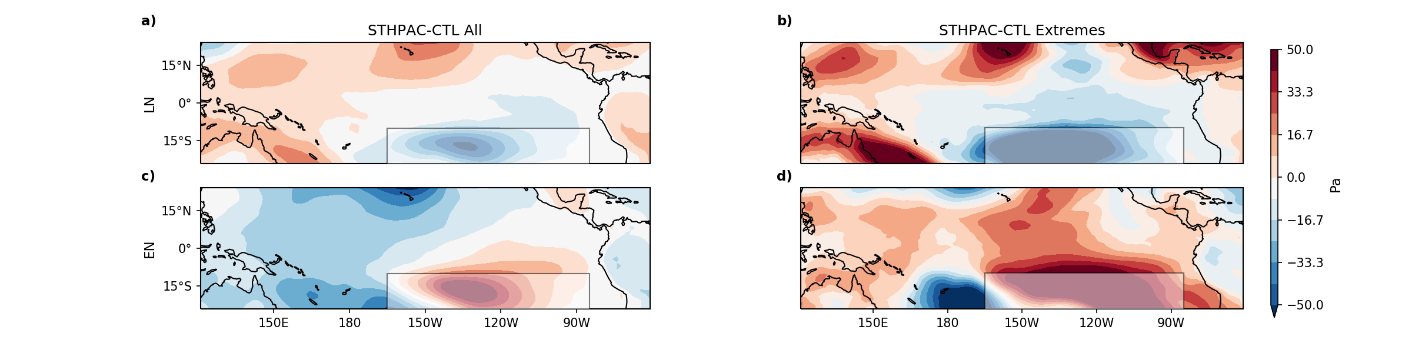


Supplementary Figure 1: Changes in sea level pressure anomalies in the Tropical Pacific. Panels show STHPAC-CTL sea level pressure anomalies averaged over (a) all La Niña years, (b) the largest 5% (extreme) La Niña years, (c) all El Niño years, and (d) the largest 5% (extreme) El Niño years.
